# A New Classification Method of Metastatic Cancers Using a ^1^H-NMR-Based Approach: A Study Case of Melanoma, Breast, and Prostate Cancer Cell Lines

**DOI:** 10.3390/metabo9110281

**Published:** 2019-11-17

**Authors:** Corentin Schepkens, Matthieu Dallons, Jonas Dehairs, Ali Talebi, Jérôme Jeandriens, Lise-Marie Drossart, Guillaume Auquier, Vanessa Tagliatti, Johannes V. Swinnen, Jean-Marie Colet

**Affiliations:** 1 Laboratory of Human Biology & Toxicology, Faculty of Medicine and Pharmacy, University of Mons, 7000 Mons, Belgium; corentin.schepkens@umons.ac.be (C.S.); Matthieu.DALLONS@umons.ac.be (M.D.); Jerome.JEANDRIENS@student.umons.ac.be (J.J.); Lise-Marie.DROSSART@student.umons.ac.be (L.-M.D.); Guillaume.AUQUIER@student.umons.ac.be (G.A.); Vanessa.TAGLIATTI@umons.ac.be (V.T.); 2 Laboratory of Lipid Metabolism and Cancer, Department of Oncology, LKI–Leuven Cancer Institute, KU Leuven, 3000 Leuven, Belgium; jonas.dehairs@kuleuven.be (J.D.); ali.talebi@kuleuven.be (A.T.); j.swinnen@kuleuven.be (J.V.S.)

**Keywords:** NMR, metabonomics, metastasis, prostate cancer, breast cancer, melanoma

## Abstract

In this study, metastatic melanoma, breast, and prostate cancer cell lines were analyzed using a ^1^H-NMR-based approach in order to investigate common features and differences of aggressive cancers metabolomes. For that purpose, ^1^H-NMR spectra of both cellular extracts and culture media were combined with multivariate data analysis, bringing to light no less than 20 discriminant metabolites able to separate the metastatic metabolomes. The supervised approach succeeded in classifying the metastatic cell lines depending on their glucose metabolism, more glycolysis-oriented in the BRAF proto-oncogene mutated cell lines compared to the others. Other adaptive metabolic features also contributed to the classification, such as the increased total choline content (tCho), UDP-GlcNAc detection, and various changes in the glucose-related metabolites tree, giving additional information about the metastatic metabolome status and direction. Finally, common metabolic features detected via ^1^H-NMR in the studied cancer cell lines are discussed, identifying the glycolytic pathway, Kennedy’s pathway, and the glutaminolysis as potential and common targets in metastasis, opening up new avenues to cure cancer.

## 1. Introduction

Cancer is a genetic or epigenetic disease resulting in altered metabolic activities compared to normal cells, including biological hallmarks such as the Warburg effect [1,2], the oxidative phosphorylation (OXPHOS) [3], and the glutaminolysis [4,5]. Such changes contribute to sustaining cancer malignancy. Due to several mutations in key oncogenes and tumor suppressor genes, cancer cells become highly demanding from a metabolic point of view, using larger amounts of carbohydrates, amino acids, and fats as energy resources to sustain rapid growth [6,7]. This results in a higher turnover of metabolites production, corresponding to intermediates or end-products of biological pathways, detectable by spectroscopic-based metabonomics approaches such as Nuclear Magnetic Resonance (NMR) or mass spectrometry (MS). During the past few years, a lot of efforts have been made to better characterize and understand the metabolic shifts in cancer cells, and this with the perspectives of identifying potential predictive diagnostic and prognostic biomarkers as well as new therapeutic targets [8,9]. Intriguingly, even at early stages of their development, tumors are able to modify the metabolic profile of peripheral biofluids (urine, blood) and tissues of the host, resulting either in fluctuations of already present metabolites or in the appearance of new ones. The signature of those cancer-related metabolites allows inferring into biochemical pathways hypothetically privileged by cancer cells and, from there, to build a specific cancer metabolome network.

Metastasis describes a phenomenon where cancer cells disseminate from a primary site to a secondary site, a critical state responsible for about 90% of deaths in cancer patients [10]. Depending on the primary cancer origin, oxygenation, pH, glucose, and other nutrients availability, metastatic cells are more likely to seed and spread in certain tissues than others [11]. For example, breast cancers preferably develop metastasis in bone, brain, liver, and lung; prostate cancers in adrenal gland, bone, liver, and lung; and melanoma in bone, brain, liver, lung, skin, and muscle [12,13,14]. From a metabolic point of view, metastatic cells are able to adapt their own metabolism depending on the secondary colonized site. For instance, metastatic breast cancers can increase their oxidative phosphorylation (OXPHOS) activity in lung and bone metastasis, whereas they are more glycolytic-orientated after invading the liver tissue [15]. Another adaptive case of breast cancer highlighted a new type of metastatic metabolism when metastases developed into brain tissue, more directed towards the consumption of glutamine and acetate in areas depleted in glucose [16,17]. 

The metabolic flexibility of metastasis complicates the handling and treatment cares, with cells adapting to their surrounding environment to support tumor growth. Therefore, identifying the preferred metabolic pathways in different types of metastatic cancer cells could open up new avenues in metastatic treatment based on metabolic interventions. Nowadays, metabonomics approaches have been successful in detaching from the one-size-fits-all treatment to adaptive treatment in the cancer field [18]. The metastatic metabolome clearly embraces this new vision of medicine, where metastatic subclasses identification will lead to greater clarity in metastasis handling. 

Proton NMR (^1^H-NMR) spectroscopy is a non-destructive technic, highly reproducible, giving an overview of the metabolome based on the simultaneous detection of a wide range of low molecular weight (LWM) metabolites. Altogether, these metabolites draw a signature that is different for each metastatic cell line. After the digitization of spectral data, individual signatures can be compared through multivariate analyses. The high demanding metabolic cell lines used in this study were selected to cover a wide range of metastatic cancer features: The MCF-7 breast cancer cell line harbors estrogen receptors whereas MDA-MB-231 was triple-negative; the bone-metastatic PC-3 cell line was TP53 mutated, whereas the LNCaP metastatic cell line was androgen-dependent; finally, the considered BRAF^V600E^-mutated metastatic melanoma were either sensitive (451-Lu, M229) or resistant (D10) to the standard of care BRAF/MEK combined inhibitors. This motley population of cell lines, which are gender-dependent or not, differ from one another in terms of oncomutation(s), tissue’s origin and metastatic dissemination site. Thus, all of them were considered in the present study in order to first obtain the most comprehensive view of the metabolome of those breast, prostatic, and melanoma metastatic cell lines and, secondly, to establish a new classification of metastases according to their metabolome. Thus, through this study, we hope to identify common biological pathways shared by the metastatic cell lines, but also some discriminating specific features, which all together could be used as potential targets for future therapeutic strategies. Hence, the importance of the biological pathways identified will be validated by the use of specific inhibitors in a cell viability test.

## 2. Results

### 2.1. ^1^H-NMR Signature from the Cellular Extracts

#### 2.1.1. Identification of Discriminant Metabolites Using Multivariate Data Analysis

**Figure 1:**
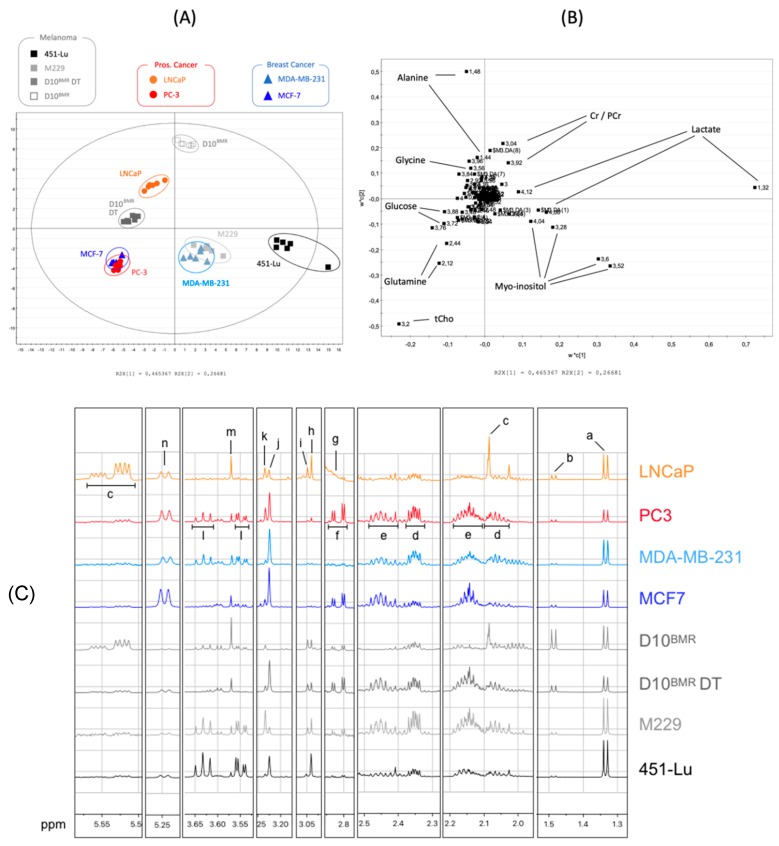
Scores plot (**A**) and loadings plot (**B**) of the partial least square (PLS-DA) analysis of the melanoma (D10^BMR^, 451-Lu, M229), prostate cancer (LNCaP, PC-3), and breast cancer (MCF-7, MDA-MB-231) ^1^H-NMR spectra of cellular extracts (R^2^X = 0.981; R^2^Y = 0.967; Q^2^cum = 0.947). (**C**) NMR spectra of the intracellular compartments reflecting the metastatic metabolome variation between the cell lines. Discriminating metabolites selected by the multivariate data analysis and the semi-quantification analysis are identified from the spectra. Spectra were normalized using the area under the curve normalization in order to show the relative intensities of discriminant metabolites, tagged with the following letters: a: Lactate; b: Alanine; c: UDP-GlcNAc; d: Glutamate; e: Glutamine; f: Aspartate; g: HEPES; h: Creatine (Cr); i: Phosphocreatine (PCr); j: Phosphocholine (PCho); k: Glycerophosphocholine (GPC); l: Myo-inositol; m: Glycine; n: Glucose.

The binned spectra (0.04 ppm stepwise) generated from the cellular extracts of melanoma (D10, 451-Lu, M229), prostate cancer (LNCaP, PC-3), and breast cancer (MCF-7, MDA-MB-231) were integrated, and the numerical values exported to an Excel table. Then, a signal normalization step was performed, dividing each 0.04 ppm length descriptor by the total area under the curve (AUC) signal of their corresponding cell line. The normalized data were next integrated into the SIMCA-P+ multivariate data analysis software in order to highlight possible metabolic differences between the different cancer cell lines. No outlier possibly arising either from experimental bias or from technical issues was detected by the principal component analysis PCA-X (Figure S1) initially applied to the dataset. A partial least square analysis (PLS-DA) was then performed on the data and returned excellent coefficients of great goodness of fit and predictability with high R^2^ and Q^2^ values (R^2^X = 0.981; R^2^Y = 0.967; Q^2^cum = 0.947). The separation observed among the experimental groups was perfectly highlighted on the scores plot (Figure 1A), where the first principal component t[1] separated the 451-Lu, MDA-MB-231, and M229 groups from the others, whereas the second principal component t[2] clearly discriminated D10^BMR^ DT, D10^BMR^, and LNCaP from the other groups. The matching loadings plot (Figure 1B) displays the most influencing descriptors, considering a variable importance in the projection (VIP) value above 1. These descriptors could be related to a subset of 10 discriminant metabolites, namely alanine, creatine (Cr), phosphocreatine (PCr), lactate, glycine, glucose, glutamine, myo-inositol, phosphocholine (PCho), and glycerophosphocholine (GPC). 

#### 2.1.2. Additional Spectra Investigations

Due to overlapping or low signal intensity, some biologically relevant information can be hidden and escape the multivariate analysis. In such cases, further information can be collected directly from the spectra using the MestRenova Peak Picking tool. Thus, two extra metabolites were detected and added to the metastatic signature thanks to additional investigations, being aspartate and UDP-*N*-Acetyl-Glucosamine. Furthermore, the resonances arising from both PCho and GPC were successfully separated from each other. The signature displayed in Figure 1C combines these additional metabolites with the previous ones identified by the PLS, and their relative intensities can be seen within the metastatic metabolomes. 

#### 2.1.3. Heatmap of the Identified Discriminant Metabolites

**Figure 2:**
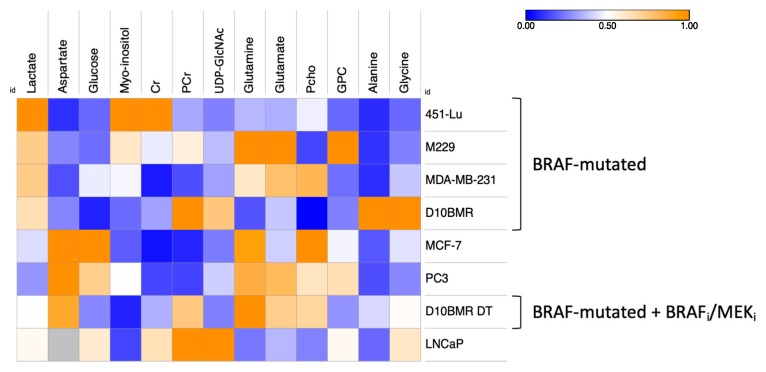
Heatmap of the 13 intracellular discriminant metabolites identified between the metastatic metabolomes. Data normalization from [0 to 1] for each metabolite enabling the metabolic signature investigation within and between the groups. For the sake of simplicity, cell lines were classified depending on their lactate and aspartate ^1^H-NMR intensities, showed via the heatmap color scale.

In order to detect common features among various cancer types, but also to find out which biological pathways were more specifically stimulated in one metastatic cancer as compared to another one, a heatmap based on ^1^H-NMR metabolites intensities was generated (Figure 2). 

Focusing on lactate and aspartate columns, an inverted relationship between these two metabolites intensities was noticed. Indeed, the cell lines harboring the highest levels of lactate also presented the lowest amounts of aspartate, and vice-versa. Interestingly, this phenomenon concerns cell lines harboring a mutation in the Raf protein, responsible for BRAF dimerization and MAPK pathway overstimulation. 

The same trend was noted for the intracellular glucose, which was handled differently depending on the metastatic profile, with the lowest amount detected in BRAF-mutated cells, indicating the highest rate in glucose turnover in those cells. 

Concerning myo-inositol, a product derived from glucose, its over synthesis was detected in three of the BRAF-mutated cell lines, and also in the PC-3 prostatic cell lines. 

Creatine and phosphocreatine, two metabolites used to store the phosphate group from the ATP energic molecule, were commonly found in the studied cancer cells lines, however, in lesser intensities than in the PC-3 and breast cancer cell lines. 

In every cell line, glutamine was detected in the intracellular space, meaning that glutamine from the media entered inside the cells, and was converted by the GLS enzyme into glutamate, a metabolite also detected in the cellular extracts. As evidence, a ^1^H-NMR experiment was carried out on metastatic cells exposed to free-glutamine media, resulting in unobservable intracellular levels of glutamine (Figure S2). The heatmap highlighted metabolites amounts quite different for glutamine and glutamate between the groups. Indeed, in some cases, only low or high amounts of both glutamine and glutamate were detected, while in the case of MCF-7 high amounts of glutamine and low amounts of glutamate were seen. 

Concerning phosphocholine and glycerophosphocholine, both metabolites were also detected in the intracellular space of the metastatic cancer cell lines. This common feature was quite intriguing and behaved separately depending on the cell lines, and with some cases, an overproduction of phosphocholine. Sometimes, both overproduced equally, or in the M229 case an overproduction of GPC only. Finally, some glycolytic characteristics of metastatic cells were also detected through the NMR analysis. First of all, UDP-GlcNAc, a metabolite synthesized from glucose, glutamine, and UTP, was detected in the D10^BMR^ and LNCaP groups, highlighting metabolic similarities in those two metastatic metabolomes. Concerning alanine, the D10^BMR^ exposed to the BRAF/MEK showed an increased production level, which was accentuated when the therapy was removed. Finally, glycine, which derives from serine metabolism, was detected at a higher level in D10^BMR^, D10^BMR^ DT, and LNCaP.

#### 2.1.4. Enrichment Analysis of the Intracellular Discriminant Metabolites

**Figure 3:**
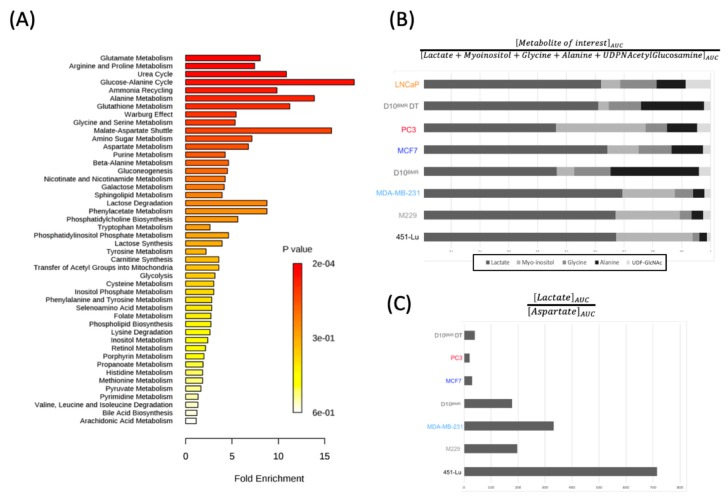
(**A**) Connection of the 13 significantly changed metabolites to relevant biochemical pathways using MSEA (**B**) Ratio of the 5 glucose-related metabolites detected through the ^1^H-NMR analysis, as an indicator of the glucose tree structure within the cells. (**C**) ^1^H-NMR lactate-to-aspartate ratio, as an estimator of the Glycolytic/OXPHOS ratio and, therefore, the energetic state of the metastatic cells.

All the 13 discriminant metabolites were then imputed into the MetaboAnalyst 4.0 online software for a Metabolite Set Enrichment Analysis (MSEA). MSEA is a strong tool used for data interpretation of metabolites set with relative concentration variations, indicating which biological pathway was more stimulated from one cell line to another. Thus, the analysis below highlighted the potential biological pathways related to the 13 identified metabolites (Figure 3A). The detected pathways were classified depending on the number of hit(s), corresponding to the number of the 13 imputed metabolites found into the identified pathways. Depending on the number of hits, the associated *p*-value can be under or above the 0.05 α–value. Vital clues from a cancer perspective can be retrieved from these MSEA analyses. 

For instance, metabolic pathways such as “glutamate metabolism”, “malate-aspartate shuttle”, and the “Warburg effect” were informative on the origin of energy resources, either from glycolysis or OXPHOS. Other information provided from “arginine and proline metabolism”, “glycine and serine metabolism”, and “amino sugar metabolism” were indicative of the various usages of glucose in cancer cells and that the glucose tree structure can be quite different depending on the cancer type.

#### 2.1.5. Data Transposition to Metabolic Ratios

A ratio (Figure 3B) was applied to five glucose-derived metabolites in order to visualize their contribution to the glucose tree structure of the three metastatic cancers metabolism. This ratio was based on the AUC (measured after integration of the corresponding spectral resonances) of five intermediates or end-products derived from glucose and detected in the ^1^H-NMR analysis, namely lactate, myo-inositol, glycine, alanine, and UDP-*N*-Acetylglucosamine. This analysis was possible because of the culture media composition, uniform between the cell lines (excepting LNCaP), and containing glucose as the main carbon source. The results highlighted two main profiles between the studied cancer cell lines. In three BRAF-mutated cell lines (451-Lu, M229, MDA-MB-231), lactate represented more than 65% of the glucose-related metabolites AUC and was associated with high myo-inositol content and lower amounts in glycine, alanine, and UDP-*N*-Acetylglucosamine. Considering the remaining cell lines, the glucose was less directed towards lactate synthesis, with lactate rates reduced from 65% to 45% and with an increase in glucose-related amino acids and UDP-*N*-Acetyl-Glucosamine. The PC-3 glucose-related metabolism was quite different from the others, with the lesser amount of lactate between all the cell lines and intensive production in myo-inositol. 

The Warburg effect and the malate-aspartate shuttle were two pathways identified by the MSEA analysis, respectively, with the following metabolites lactate and aspartate. Lactate, the end-product of glycolysis, indicated that ATP production was favored via the glycolytic pathway, whereas aspartate, a metabolite part of the malate-aspartate shuttle used for translocating electrons across the mitochondria membrane indicated ATP production through OXPHOS. Therefore, it would be advantageous to know how cancer cells used glucose for ATP synthesis. In this respect, the lactate-to-aspartate AUCs ratio was indicative. The calculated ratio shown in Figure 3C revealed two trends. The first one is composed of the D10^BMR^ DT, PC3, and MCF-7 cell lines that harbored a higher level of aspartate and a lower level of lactate, resulting in a small Glycolytic/OXPHOS ratio. The ratios of the D10BMR, MDA-MB-231, 451-Lu, and M229 took the opposite direction and were increased significantly, with levels >100. The 451-Lu was clearly the most glycolytic cell line based on the ratio analysis, with a value above 700. This finding is in adequacy with the PLS model described in Figure 1, where the 451-Lu model was also incriminated in lactate overproduction.

### 2.2. Extracellular Compartments Analysis

**Figure 4:**
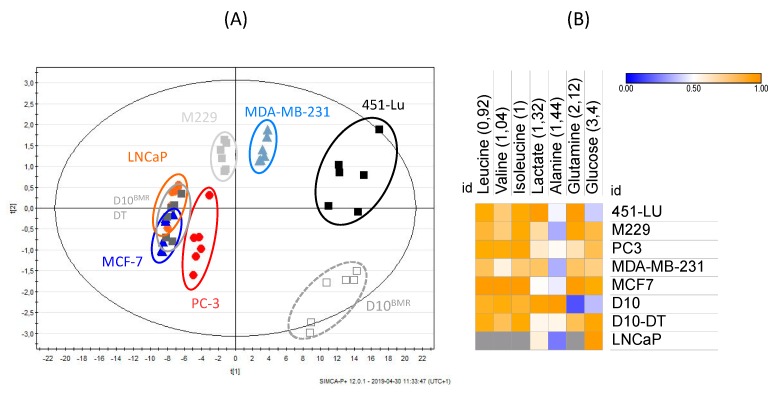
Scores plot (**A**) and loadings plot (**B**) of the PLS-DA analysis of the melanoma (D10^BMR^, 451-Lu, M229), prostate cancer (LNCaP, PC-3), and breast cancer (MCF-7, MDA-MB-231) extracellular compartment ^1^H-NMR spectra (R^2^X = 1; R^2^Y = 0.99; Q^2^cum = 0.97).

A supervised multivariate data analysis (PLS-DA) was also carried out on the ^1^H-NMR spectra of the culture media, returning high R^2^ and Q^2^ values (R^2^X = 1; R^2^Y = 0,99; Q^2^cum = 0,97). Groups separation was highlighted on the scores plot (Figure 4A), where the first principal component t[1] separates the 451-Lu, MDA-MB-231, M229 and D10^BMR^ groups from the others. This separation is described on the corresponding loadings plot (Figure S3) and was mainly depending on glucose consumption and lactate secretion, and both increased in the 451-Lu, MDA-MB-231, M229, and D10 groups. The second principal component t[2] involved the ability of the cancer cells to secrete alanine, increased in the D10^BMR^ group. Further analyses were performed on all DMEM-exposed groups but LNCaP, and added to the heatmap in Figure 4B. Thus, the variations in amino acid contents in the media of the studied metastatic cell lines revealed which ones relied more on amino acid consumption. The BRAF-mutated cell lines seemed more dependent on the essential amino acids leucine, isoleucine, and especially valine, while the D10^BMR^ and PC-3 cell lines were eager on glutamine consumption to supply their intermediary metabolism.

### 2.3. Metabolic Inhibition of the Glycolytic, Glutamine, and Choline Pathways 

**Figure 5:**
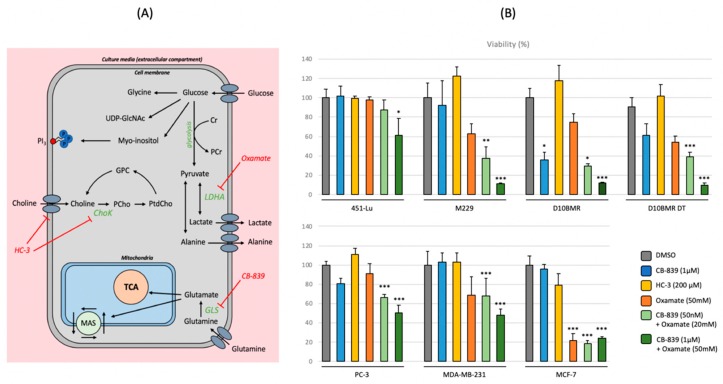
(**A**) Overview of the metastatic metabolome ^1^H-NMR signature, including the established mechanism of the following metabolic inhibitors: Oxamate, CB-839, and Hemicholinium-3 (HC-3). (**B**) Crystal violet staining absorbance (570 nm) of the metastatic cell lines displayed as a percentage. For each condition, a number of 12 wells (*n* = 12) were performed. Statistical analysis of the data was performed using the non-parametric Kruskal—Wallis test. Significant differences of experimental groups compared to the control (DMSO) group were tagged as follow: * *p* < 0.05, ** *p* <0.01, *** *p* < 0.001.

In order to verify whether the biochemical pathways identified by the metabonomics approach were indeed critical for cancer cell survival and growth, specific chemical inhibitors were tested on each cell line and cell viability measured, except for LNCaP, which were not appropriate cells for such test due to their poor adherence properties. Thus, three inhibitors named CB-839, Hemicholinium-3 (HC-3), and Oxamate were used to, respectively, impact the glutaminolysis, the choline metabolism, and the lactate production. The established mechanisms of the considered inhibitors are displayed in Figure 5A, combining with the previously identified pathways from the ^1^H-NMR analysis. Viability results are merged in Figure 5B, with the inhibitors used separately or in combination for synergistic effect. Both Oxamate and CB-839 were able to reduce cell viability as single agents. Therefore, the combination of CB-839 and Oxamate was next investigated, with respectively a small and high dose combination of both compounds to evaluate a potential synergistic effect. The high dose combination included the dose of Oxamate and CB-839 used as a single agent, whereas the small dose combination was 2.5-fold less concentrated in Oxamate and 20-fold less concentrated in CB-839. The small dose combination was able to significantly reduce the cell viability in the considered metastatic cell lines, except in the 451-Lu case. Increasing the dose of both compounds resulted in all cases in a sustained and statistical decrease of cell viability.

## 3. Discussion

### 3.1. ^1^H-NMR Identification of Metastatic Metabolome Subclasses 

Based on the lactate and aspartate intracellular levels, two metastatic subclasses were identified in terms of ATP synthesis, either more glycolytic- or more OXPHOS-oriented. As expected, the BRAF-mutated cell lines presented the most glycolytic-orientated NMR signatures, with a higher amount in lactate, the end-product of glycolysis, in both intra- and extracellular compartments [19], and a lower amount in intracellular aspartate that normally reflects the malate-aspartate shuttle activation for ATP production via the oxidative phosphorylation [20,21]. Indeed, BRAF has been incriminated in the MAPK pathway overstimulation, resulting in the downstream stabilization of the hypoxic inducible transcription factor-1alpha (HIF-1α) in BRAF^V600E^ melanoma [22], a transcription factor also overexpressed in the BRAF-mutated MDA-MB-231 breast cancer cell lines [23]. It is well known that HIF-1α increases the glycolytic activity of cancer cells, resulting in the overexpression of glycolytic key enzymes like hexokinases (HK) or lactate dehydrogenase (LDH-A), and glucose and lactate transporters such as the GLUT-1, GLUT-3, and MCT-4 [24,25]. Therefore, it may be assumed that metastatic cells harboring glycolytic-preponderant mutations naturally direct their ATP production towards glycolysis. This metabolic feature is in adequacy with the calculated lactate/aspartate ratio, with BRAF-mutated metastasis being more glycolytic-orientated [26]. Interestingly enough, the BRAF inhibition with the BRAF_i_/MEK_i_ therapy redirects the ratio in the D10^BMR^ cell lines from glycolytic-orientated to OXPHOS-orientated, meaning that BRAF inhibition may switch the ATP production metabolism. Furthermore, the consumption of extracellular glucose and its intracellular handling also reflected the two metabolic profiles, increased in BRAF-mutated cell lines thanks to glycolytic preferred activation.

Phosphocreatine (PCr) is synthesized from creatine (Cr) by creatine kinase (CK) [27], a shuttle system identified in cancer cells to store phosphate groups from ATP and enabling its restitution in energy-consuming processes such as in some steps of glycolysis [28]. Besides, the CK enzyme has been incriminated in cell cycle regulation and also cell mobility, with metastatic cells harboring a high amount of CK [28]. The ^1^H-NMR approach revealed two metabolites related to this energetic shuttle, namely creatine and phosphocreatine. Three metastatic cell lines (PC-3, MCF-7, MDA-MB-231) displayed reduced intensities for those metabolites in their NMR-based metabolomes. This observation suggests the limited use of this metabolic shuttle in those cell lines. Therefore, this feature could be used to classify metastatic cells since some of them preferably rely on the creatine shuttle. In addition, such a metabolic singularity could be targeted by specific inhibitors of the creatine shuttle [29]. For those reducing the use of the creatine shuttle, an element of the answer could be provided from the Kennedy’s pathway, part of the choline metabolism. It is well known that in cancer cells the choline metabolism is deregulated at two different levels. Firstly, the total choline content is increased as compared to normal cells and, secondly, a modulation of the phosphocholine (PCho) to glycerophosphocholine (GPC) ratio can be observed, with an increase in PCho and a decrease in GPC [30,31,32]. Phospholipase C, an enzyme contributing to the choline metabolism, catalyzes the hydrolysis of phosphatidylcholine into PCho and diacylglycerol, a second messenger that activates the phosphokinase C (PKC) responsible for cancer cells proliferation, survival, and RAF activation [33,34]. Interestingly, the metabolome of PC-3, MCF-7, and MDA-MB-231 included the highest level of PCho, together with the lowest amount in tCr. One can assume that either the creatine shuttle or the choline metabolism could sustain the metastatic phenotype. Hence, they should be considered and handled differently depending on the metastatic metabolic direction. 

Cancer cells are highly addicted to glucose uptake and metabolism, which can be consumed through different pathways depending on their own genome mutations. Here, some glucose-related metabolites detected in the metastatic ^1^H-NMR signature gave an overview of this differential glucose handling. BRAF-mutated cell lines showed a common glucose-related profile clearly separated from the others, orientated in both lactate and myo-inositol production, indicating a glucose use for ATP and phosphoinositides productions, respectively. As discussed, all metastatic cell lines produced detectable levels of lactate, indicating sustained anaerobic glycolysis. However, BRAF-mutated cell lines showed the greatest lactate levels as a potential response to MAPK overstimulation, HIF-1-α stabilization, and sustained glycolysis. In addition, BRAF-mutated metastases overproduced myo-inositol, a precursor used in cancer cells for the synthesis of phosphoinositides, messengers involved in the phosphoinositide signaling system. These lipids family are located in the inner membrane of the cells and are involved in pro-tumoral key protein regulation such as PTEN and AKT, resulting in cell survival, proliferation, and growth [35]. Interestingly, this feature was also detected in the PC-3 cell line, less glycolytic-orientated but showing a high amount of myo-inositol. Focusing now on the other cell lines, a larger use of glucose was detected, with a glycolytic tree orientated for lactate, amino-acids (glycine, alanine), and UDP-GlcNAc (D10^BMR^, LNCaP) production. Glycine is produced from glucose through serine metabolism, via the action of phosphoglycerate dehydrogenase (PHGDH) [36]. PHGDH was shown overexpressed in some tumors, and its downregulation impaired cancer growth [37]. Considering the alanine increase, it is correlated to an increase of protein synthesis in those cells [36]. It is not unexpected to find the D10^BMR^ cell lines in this category, because BRAF_i_ resistance has been incriminated in the metabolic switch of melanoma cells, with an increase in oxidative metabolism and glutamine dependency [38]. Finally, the detection of UDP-*N*-Acetylglucosamine, a metabolite derived from glucose and glutamine, in LNCaP and D10^BMR^ cell lines indicated a post-translational ability of the metastatic cells, to add a *N*-acetylglucosamine group on serine and threonine residues of key proteins, promoting migration and invasion [39]. A recent study showed that inhibiting the UDP-GlcNAc production, by targeting the O-GlcNAc transferase, decreases cell proliferation and invasion in LNCaP [40]. As a result, the ^1^H-NMR signature may give some clues on how glucose is preferentially handled in different metastases, for phosphoinositides synthesis in glycolytic-orientated cells, and amino acids synthesis in OXPHOS-orientated cells. In addition, the production and detection of UDP-*N*-Acetylglucosamine indicate an O-glycosylation of key proteins promoting metastasis invasion.

### 3.2. Disrupting Metastatic Metabolome Using Metabolic Inhibitors

Our experimental approach also picked up some biochemical pathways shared by the considered metastatic cancer cells, such as those involving the glucose-related metabolites, glutamine, and glutamate, and choline-related metabolites. Although the relatively low sensitivity of 1H-NMR (0.1 mM) is criticizable, nevertheless its ability to pinpoint the main metabolic hallmarks of a specific cell type makes it a suitable technic for cancer investigation. In this study, the NMR results highlighted the Kennedy’s pathway, the glycolysis, and the glutaminolysis as three relevant pathways showing different relative intensities according to the genetic profiles of the metastatic cancer cells.

Enhanced glycolysis enzymes have been strongly correlated as a cancer hallmark, underlying the high-dependency of cancers to glucose uptake and metabolism. Indeed, GLUT-1 and GLUT-3 transporters presenting higher expression and activity (10–12 fold higher) in cancer cells as compared to healthy tissues [41]. The end-product of this enhanced glycolysis is lactate, originating from the conversion of pyruvate thanks to the lactate dehydrogenase A activity (LDH-A). The higher production of lactate supports the cancer malignant status by restoring the NAD+ cellular pool and stimulating glycolysis in cancer cells. In addition, lactate also promotes angiogenesis, the escape from the immune system, and the acidification of the microenvironment [42]. As a result, inhibiting the LDH-A activity with the small inhibitor Oxamate has already been shown to weaken cancer metabolism [42,43]. Oxamate was, therefore, selected in our strategy as the first metabolic inhibitor.

Glutamine, one of the most blood circulating amino acids, is overused by cancer cells to produce antioxidants, lipids through the de novo lipogenesis process, as well as purines and pyrimidines [5]. Glutamine penetrates cancer cells using the SLC1A5 transporter, which is upregulated in many cancers such as non-small cell lung cancer (NSCLC), breast cancer, and brain tumors [44,45]. Next, glutamine is converted into glutamate thanks to the GLS enzyme activity, which is also upregulated in many cancers [46]. Therefore, the GLS presents itself as a key element in the glutaminolysis process, targetable with small inhibitors such as CB-839 [4,5]. Regarding its anti-glutaminolysis activity, CB-839 was, therefore, selected in our strategy as the second metabolic inhibitor.

The choline metabolism was also incriminated in our study, with both detection of phosphocholine (PCho) and glycerophosphocholine (GPC). A correlation between the choline metabolism enzymes and the choline metabolism metabolites have already been described in cancer. Higher amounts of tCho metabolites were detected in both prostate and breast cancer tissues, with respect to healthy ones, and associated with an increase in key enzyme activity and/or expression [31]. It appears that the ChoK enzyme, responsible for choline transformation into PCho, is strongly correlated to cell proliferation and human tumors [47]. As it turns out, Hemicholinium-3 (HC-3), a known inhibitor of the ChoK enzyme [48], was considered as the third inhibitor in our strategy.

In this regard, a cell viability strategy was investigated to jeopardize metastatic cells by using specific inhibitors of critical cancer cell pathways. Interestingly, our findings suggest that blocking simultaneously both glutaminolysis and lactate production, using CB-839 and Oxamate, disrupts metastatic cell viability. Nowadays, these inhibitors are mainly used alone, or in combination with the standard of care in cancer treatments. However, a few promising studies investigated the synergistic combination of these inhibitors and provided encouraging results, such as the couple CB-839/3-BP that demonstrated positive synergistic effects in tuberous sclerosis mice [49].

This study provides a novel metastatic metabolome classification, highlighting common metabolic shares and differences between prostate, breast, and melanoma metastatic cell lines. The overview of the metabolome enabled the selection of three targetable pathways, being the glycolysis, the glutaminolysis, and the Kennedy’s pathway. As it turns out, the viability test validated the combined glutaminolysis and glycolysis inhibition, opening up an interesting approach in the metastatic metabolome disruption.

## 4. Materials and Methods 

### 4.1. Cell Lines and Culture 

Melanoma cell lines were composed of M229, 451-Lu and D10^BMR^ cell lines, prostate cancer was represented by the PC-3 and LNCaP cell lines, and breast cancer by the MCF-7 and MDA-MB-231 cell lines. The melanoma cell lines used in this study (M229, 451-Lu, D10BMR) were kindly provided from the laboratory of Lipid Metabolism and Cancer (KUL-Belgium), and used in the following publication (https://www.nature.com/articles/s41467-018-04664-0). The PC-3 (ATCC^®^ CRL-1435™), LNCaP (ATCC^®^ CRL-1740™), MDA-MB-231 (ATCC^®^ HTB-26™) and MCF-7 (ATCC^®^ HTB-22™) cell lines were purchased from ATCC. All cells, except LNCaP, were grown in 75 cm^2^ flask using DMEM High-Glucose (ThermoFisher 11960, Merelbeke, Belgium) supplemented with 4 mM of Glutamine (ThermoFisher), 10% of fetal bovine serum (Gibco Lot 42G8378K), 100 U/mL of penicillin, and 100 μg/mL of streptomycin (ThermoFisher). LNCaP condition was slightly different with the use of RPMI 1640 (ATCC 30-2001) instead as a growth media. Concerning the D10^BMR^ melanoma cell line, two conditions were included depending on the presence or absence of the Dabrafenib/Trametinib therapy (Selleckchem). Thus, the D10^BMR^ DT condition corresponded to the cells that were grown in the previous described media with an addition of 0.4 μM Dabrafenib and 0.1 μM Trametinib dose, whereas D10^BMR^ were grown with the therapy and then deprived of it during 72 h for metabolic changes observations. 

### 4.2. Metabolic Inhibitors and Viability Test

The following metabolic inhibitors were used in this study: CB-839 (Cayman Chemical) solubilized in DMSO, Hemicholinium-3 and Oxamate (Sigma-Aldrich) directly solubilized in the culture medium. For viability testing, cells were first seeded in a 96-well plate (4-6 k cells/well) and grown for 4 days in 10% FBS media. Then, the media were removed and replaced by fresh media containing the metabolic inhibitor(s) for 72 h. After incubation, cells were washed twice with PBS and fixed with glutaraldehyde 4%. The staining was then performed using a 1% crystal violet solution, and cell wall permeabilized using Triton. A number of *n* = 12 wells were performed for each condition, and absorbance was read at 570 nm. 

### 4.3. ^1^H-NMR Samples Collection and Extraction

Every cell line was grown to confluency before sample collection, with *n* = 6 samples per group. Culture media were replaced by fresh media with 10% FBS to standardize the NMR analysis 24 h before collecting the samples. The 24 h culture media were removed from each flask and stored at −20 °C. The remaining cells were washed twice with 7 mL cold D-PBS (Sigma-Aldrich), quenched using 3 mL of cold methanol, and then collected using a scraper. Cells metabolism was de novo quenched by immersing the cell pellet into liquid nitrogen for a few seconds before storing at −80 °C. A methanol: water: Chloroform 1:0.9:1 (3 mL) extraction was carried out on the cell pellet to separate polar metabolites from macromolecules. The polar phase was dried using a SpeedVacuum and was stored at −80 °C before analysis. 

### 4.4. ^1^H-NMR Spectroscopy

The intracellular dried samples were resuspended in 700 μL phosphate buffer (0.2 M Na_2_HPO_4_/0.04M NaH_2_PO_4_, pH 7.4) prepared in a mixture of H_2_O/D_2_O (80:20; v:v). The samples were centrifuged at 13,000 g for 10 min. 50 μL of a 7 mM solution of 3-trimethylsilyl propionic-2,2,3,3-d4 acid (TSP) reference prepared in 100% deuterium oxide was added to 650 μL of each supernatant. Finally, 700 μL of this final mixture was transferred into 5 mm NMR tubes prior to analysis. For the culture media preparation, 500 μL of each sample were mixed with 250 μL of phosphate buffer. The samples were prepared as described above, with an addition of 14 mM TSP solution. Acquisition of the ^1^H-NMR spectra were processed on a Bruker 600-MHz Advance spectrometer for ^1^H, using the NOESYPRESAT-1D pulse sequence. The same acquisition method was applied to the samples of cellular extracts and culture media using 256 scans.

### 4.5. Spectra Processing and Multivariate Data Analysis

^1^H-NMR spectra were processed using the MestreLab Research 10.0.2 software (Mestrelab Research, S.L, Santiago de Compostela, Spain). Phases and baseline of each spectra were corrected using the automatic and manual software tools. The water peak region ranging from 4.20 to 5.50 ppm was excluded, and spectra intensities were calibrated to the TSP chemical shift arbitrary fixed at 100. Spectra were binned in small subregions of 0.04 ppm width called descriptors, which gave rise to 220 descriptors for each spectrum. Next, the area under the curve (AUC) was calculated for each descriptor in all spectra to obtain numerical data. Each descriptor value was divided by the total area of the spectrum for normalization. Data were then processed for multivariate data analysis using the software SIMCA-P+ 12.0 (Umetrics, Umeå, Sweden). Principal Component Analysis (PCA) followed by a Partial Least Square Discriminant Analysis (PLS-DA) were carried out on the dataset. Possible group separations were observed in the scores plot and descriptors responsible for such separation on the loadings plot. Only descriptors with a VIP >1 were considered, and their corresponding metabolites were identified. To evaluate the quality of the model, two parameters were considered, R^2^, which corresponds to the « goodness of fit parameter » and explains variation in the data, and Q^2^, which is the « goodness of prediction parameter » and represents the predictive power of the model. Validation was carried out for the PLS model reliability using two tests: A permutation test followed by a cross-validation analysis of variance test (CV-ANOVA). Discriminant descriptors were correlated to metabolites using the Chenomx NMR suite software (version 8.1.1) and the Human metabolome database (HMDB). Because of the descriptor size of 0.04 ppm that can contain several metabolites chemical shifts, and also metabolites that have a low intensity, the multivariate data analysis was not able to detect all of the discriminant metabolites. A semi-quantification comparison of the spectra in the MestRenova software was then processed to find these new metabolites that can escape from our first analysis. 

### 4.6. Metabolic Signature Validation, Heatmap, and Enrichment 

The Peak Peaking tool of the MestRenova software was able to detect and precisely calculate the area under the curve (AUC) of all the peaks from each spectrum and was used for the metabolic signature validation. For each discriminant metabolite detected in the previous step, the AUC of one isolated chemical shift, previously normalized by the total AUC of each spectrum, was calculated using the Peak Picking tool in order to obtain numerical data. These numerical data were then processed in the R statistical software, using two non-parametric simultaneous tests on the data. Basically, a Kruskal—Wallis test followed by a Dunn test was performed. The Holm method adjustment was used in this validation process and applied to *p*-values. Discriminant identified metabolites were converted to a heatmap in order to better visualize the metabolic signature between the cell lines. The heatmap was generated using the Morpheus online software. To identify the most changed pathways between the cell lines, a metabolic enrichment was performed on the discriminant metabolites using the online available software Metaboanalyst 4.0. The Homo Sapiens Pathway Library was selected as a reference, and the pathway analysis was investigated based on the *p*-values from pathway enrichment analysis and pathway impact values.

## Supplementary Materials

The following are available online at https://www.mdpi.com/2218-1989/9/11/281/s1, Figure S1: Principal component analysis (PCA-X) of the intracellular metastatic compartments of the different cancer cell lines used in this study. Figure S2: Intracellular space of the 451-Lu cell line either grown into a glutamine-containing (black) or glutamine-free (red) medium. Figure S3: Scores Plot (A) and Loadings Plot (B) of the PLS-DA analysis of the melanoma (D10^BMR^, 451-Lu, M229), prostate cancer (LNCaP, PC-3), and breast cancer (MCF-7, MDA-MB-231) ^1^H-NMR spectra culture media. Figure S4: Intracellular (A) and extracellular (B) ^1^H-NMR spectra of the 451-Lu cell line. Figure S5: Intracellular (A) and extracellular (B) ^1^H-NMR spectra of the M229 cell line. Figure S6: Intracellular (A) and extracellular (B) ^1^H-NMR spectra of the D10^BMR^ cell line. Figure S7: Intracellular (A) and extracellular (B) ^1^H-NMR spectra of the D10^BMR^ DT cell line. Figure S8: Intracellular (A) and extracellular (B) ^1^H-NMR spectra of the MCF-7 cell line. Figure S9: Intracellular (A) and extracellular (B) ^1^H-NMR spectra of the MDA-MB-231 cell line. Figure S10: Intracellular (A) and extracellular (B) ^1^H-NMR spectra of the PC-3 cell line. Figure S11: Intracellular (A) and extracellular (B) ^1^H-NMR spectra of the LNCaP cell line.
