# Supplementary material for: A New Classification Method of Metastatic Cancers Using a 1H-NMR-Based Approach: A Study Case of Melanoma, Breast, and Prostate Cancer Cell Lines
Source: Metabolites. 2019 Nov 17;9(11):281. doi: 10.3390/metabo9110281 (PMC6918216; doi:10.3390/metabo9110281)

Figure S1. Principal component analysis (PCA-X) of the intracellular metastatic compartments of the different used cancer cell lines.

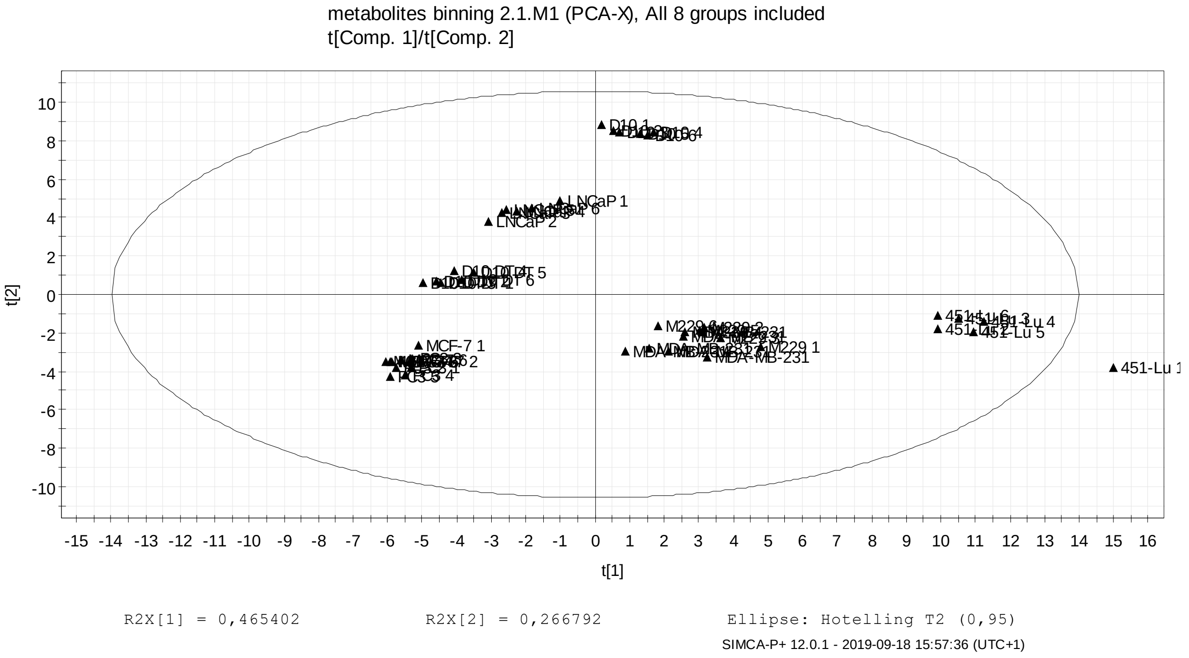

R^2X: 0,985 ; Q^2cum: 0,937

Figure S2. Intracellular space of the 451-Lu cell line either grown into a glutamine-containing (black) or glutamine-free (red) medium.

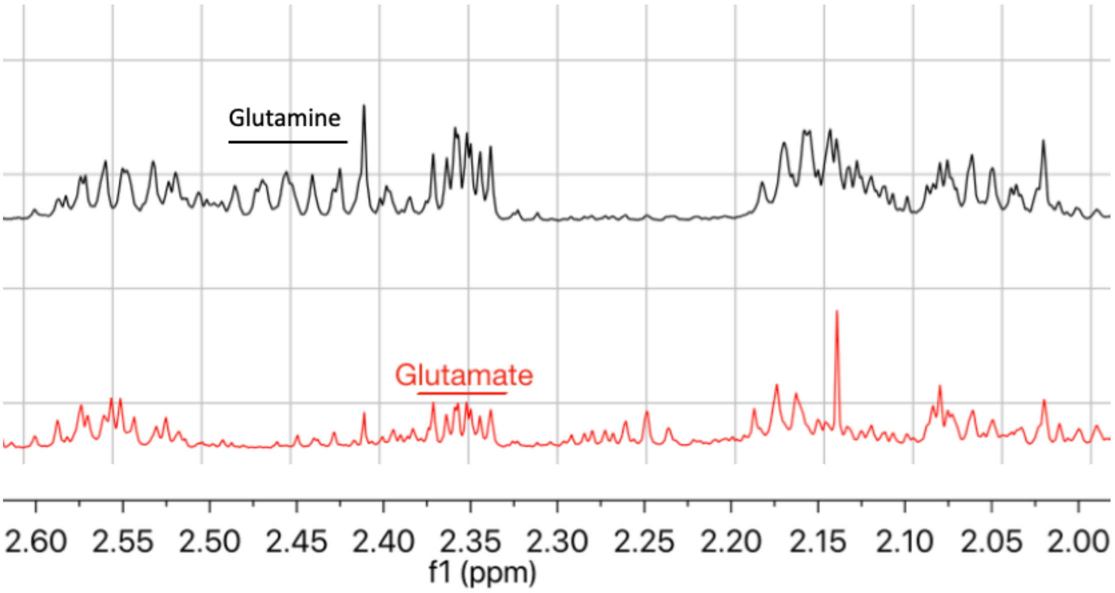

Figure S3. Scores Plot (A) and Loadings Plot (B) of the PLS-DA analysis of the melanoma (D10BMR, 451-Lu, M229), prostate cancer (LNCaP, PC-3) and breast cancer (MCF-7, MDA-MB-231) ^{1}H-NMR spectra culture media.

(A) (B)

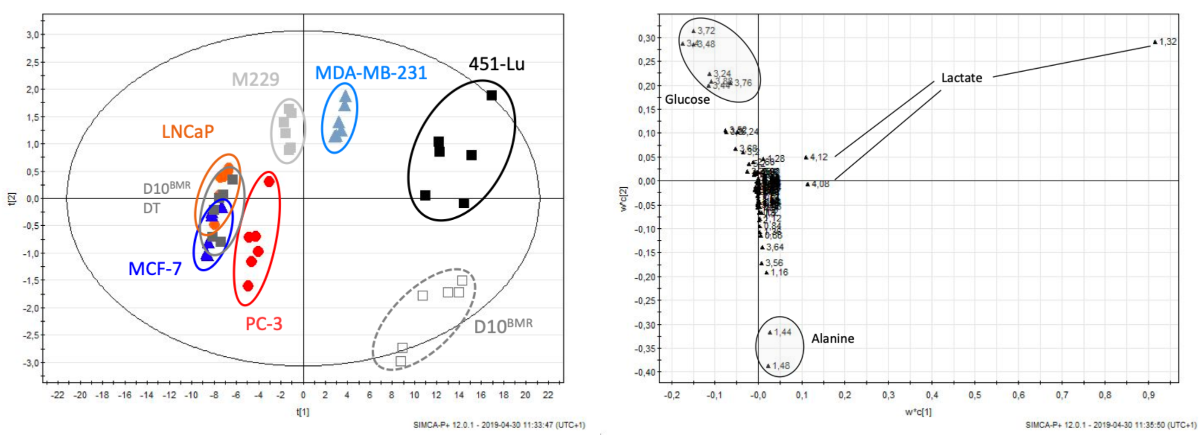

Figure S4. Intracellular (A) and extracellular (B) ^{1}H-NMR spectra of the 451-Lu cell line.

(A)

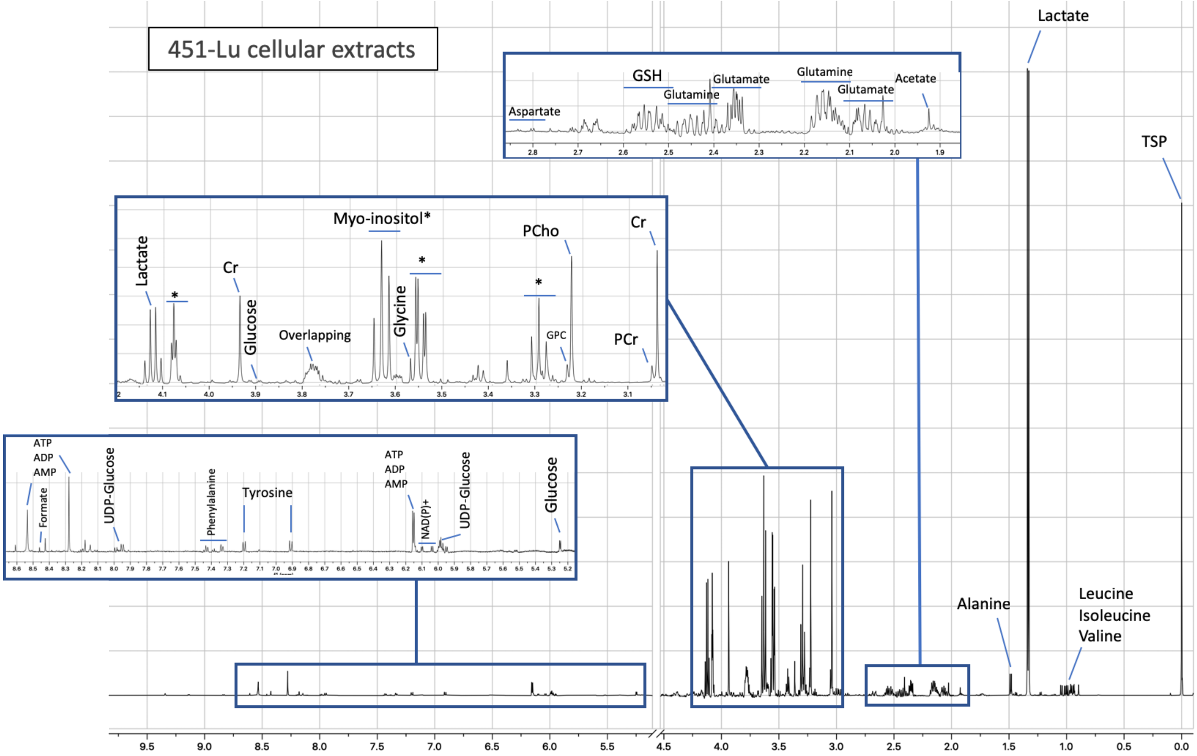

(B)

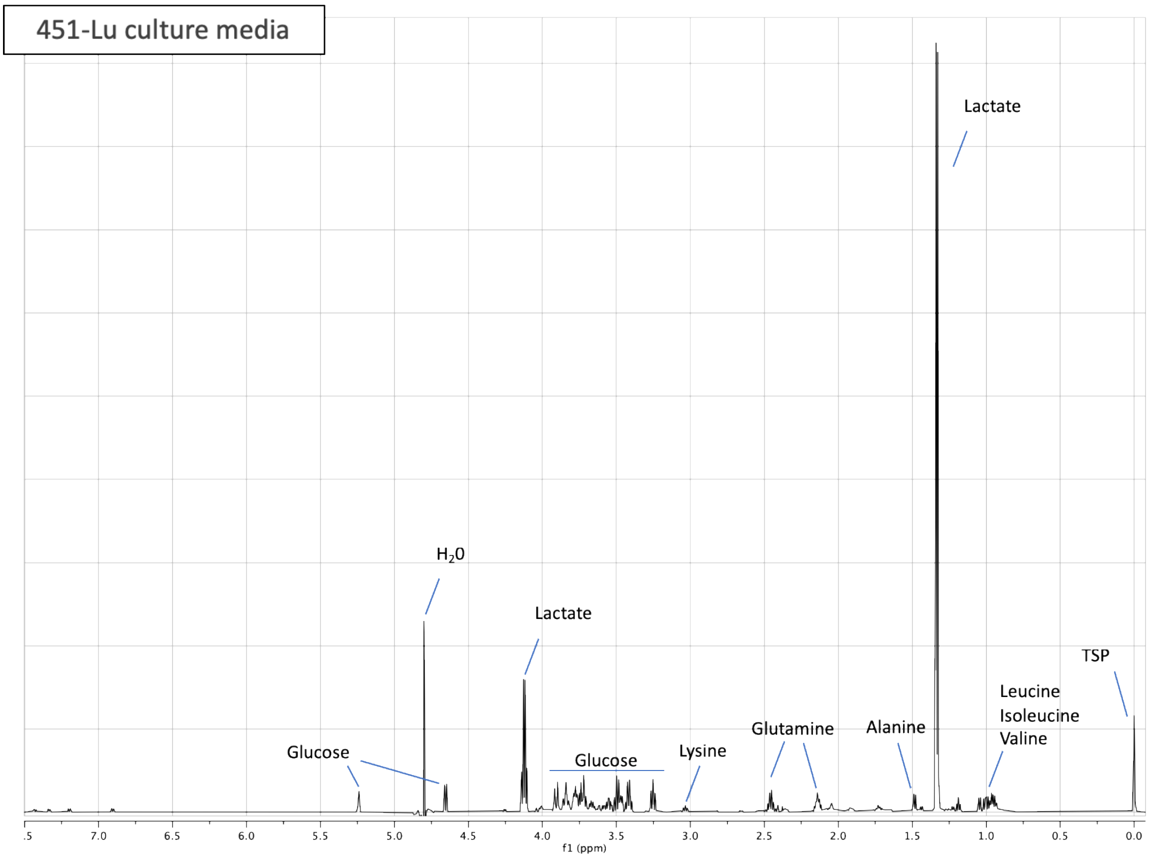

Figure S5. Intracellular (A) and extracellular (B) ^{1}H-NMR spectra of the M229 cell line.

(A)

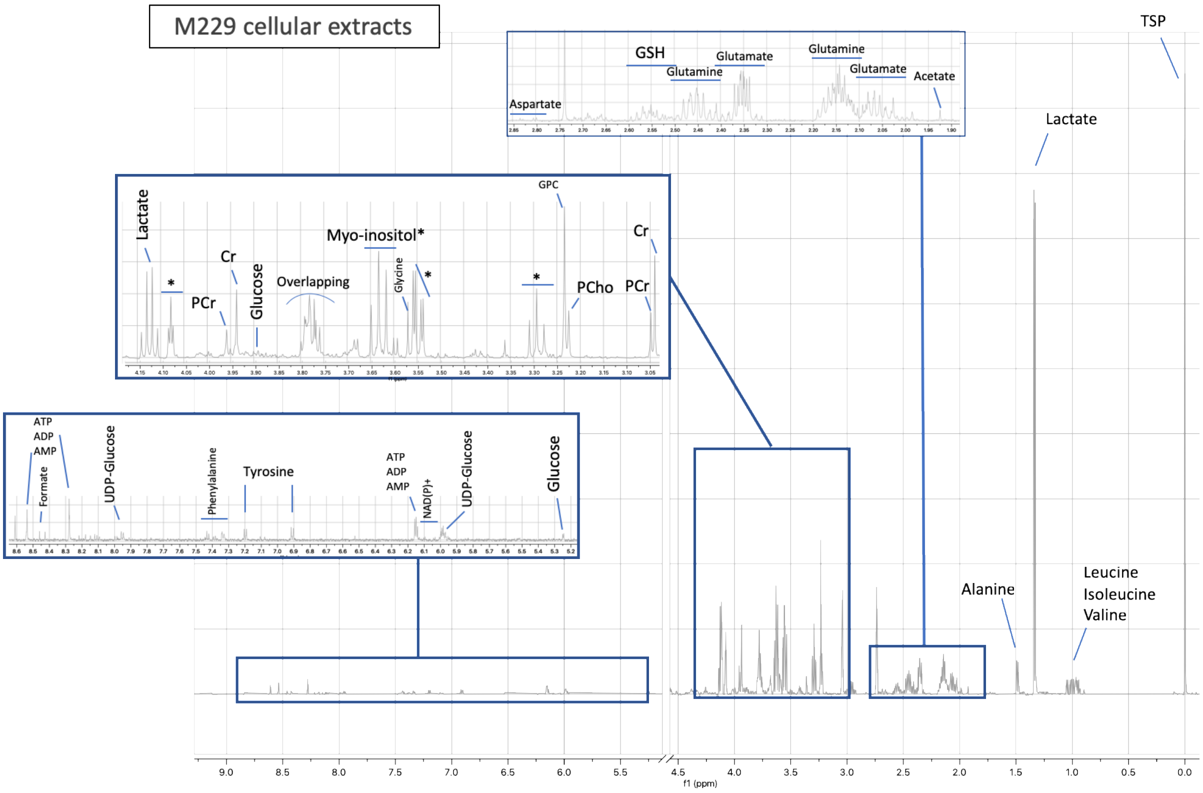

(B)

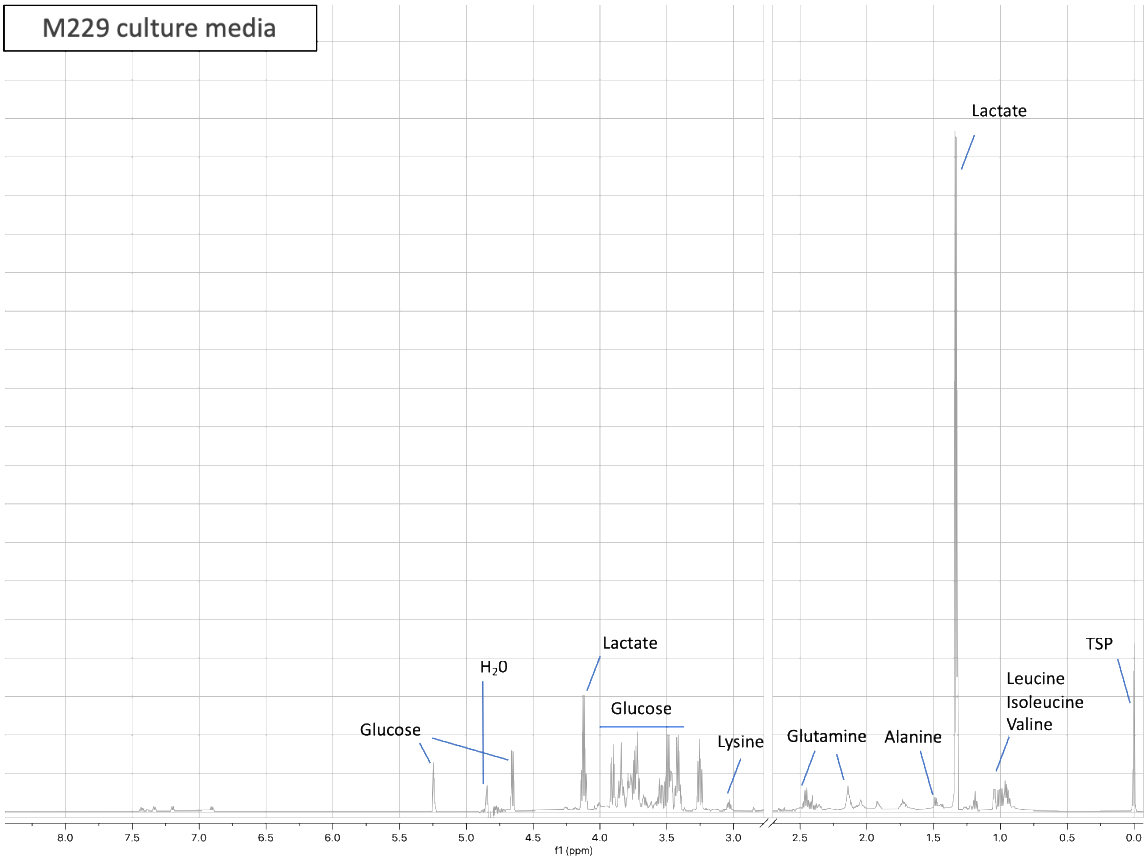

Figure S6. Intracellular (A) and extracellular (B) ^{1}H-NMR spectra of the D10^{BMR} cell line.

(A)

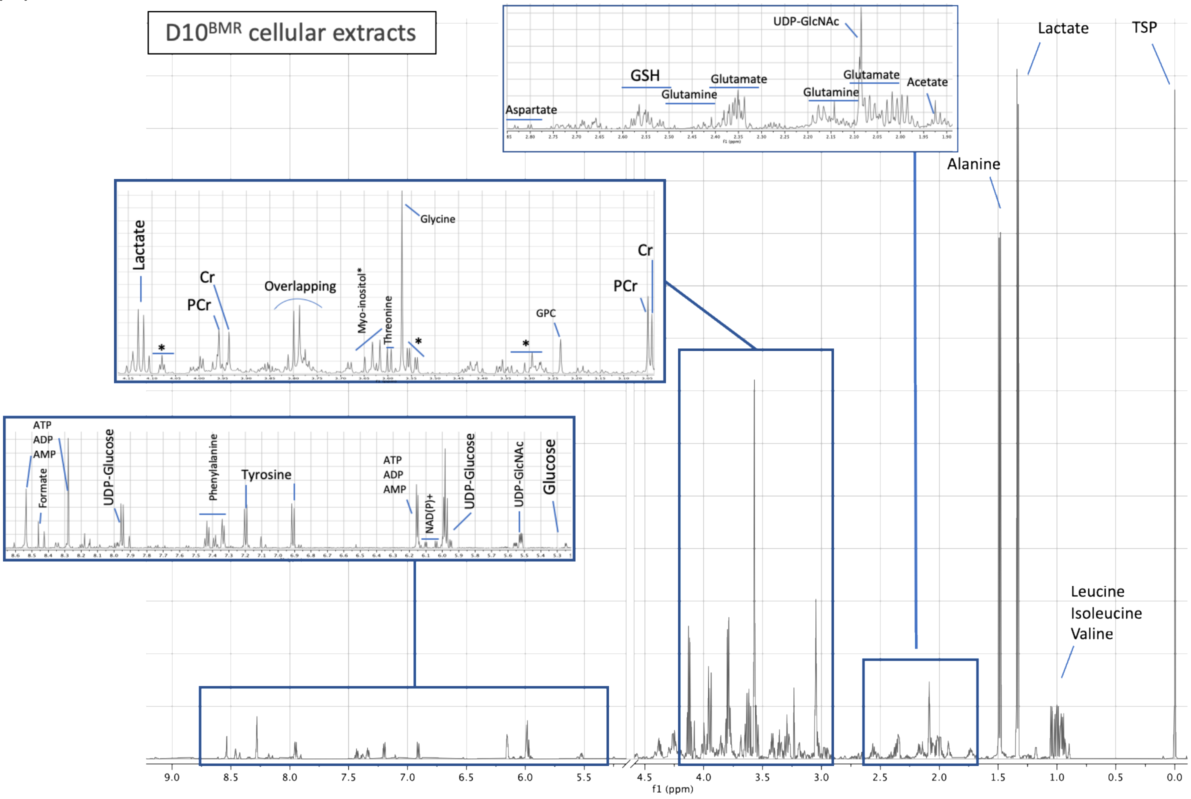

(B)

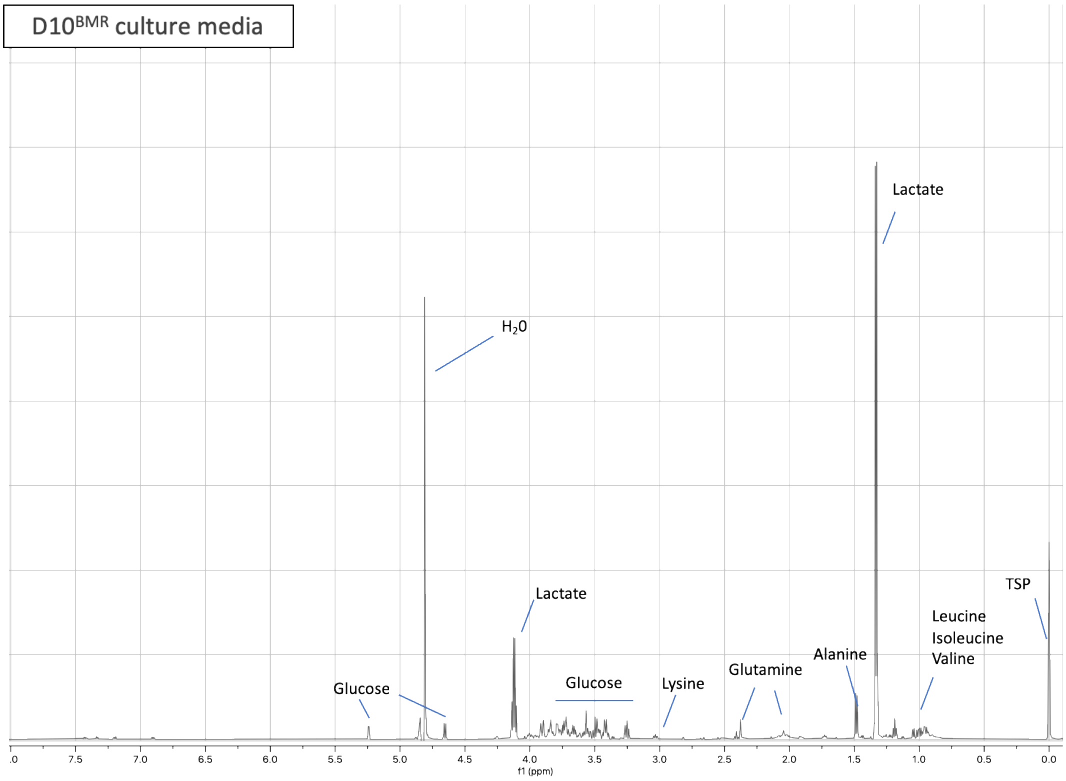

Figure S7. Intracellular (A) and extracellular (B) ^{1}H-NMR spectra of the D10^{BMR} DT cell line.

(A)

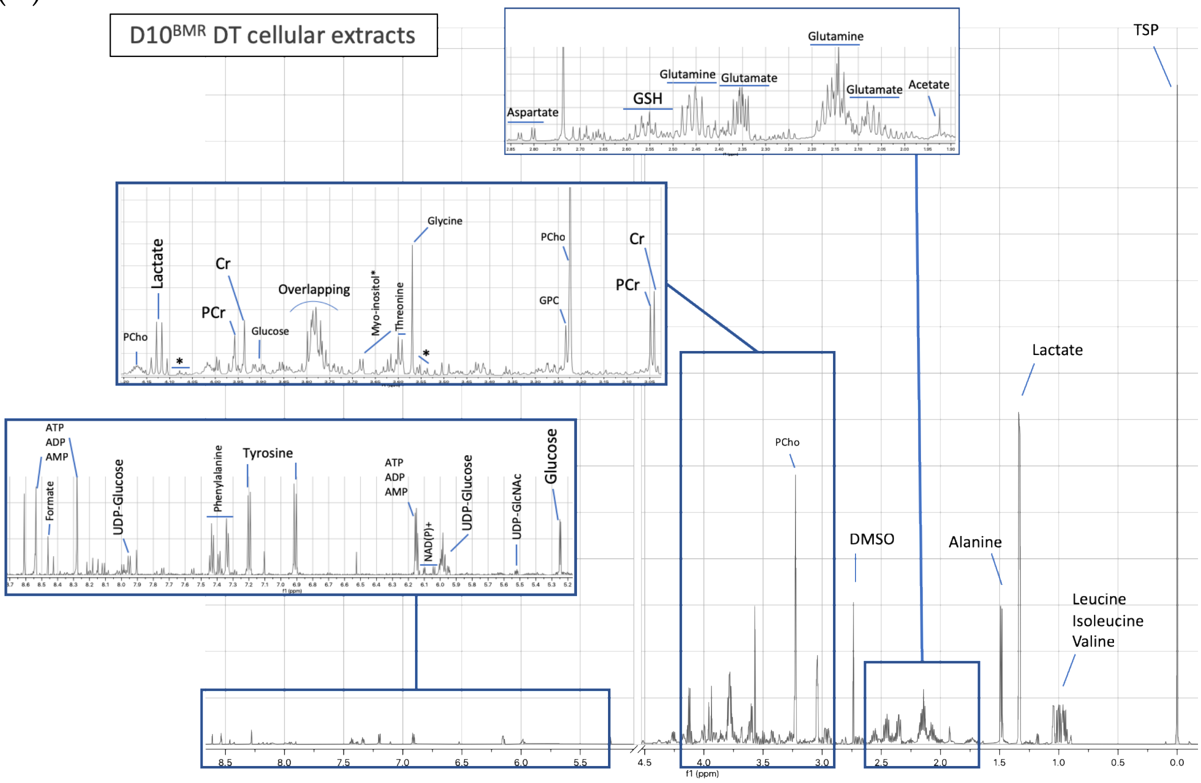

(B)

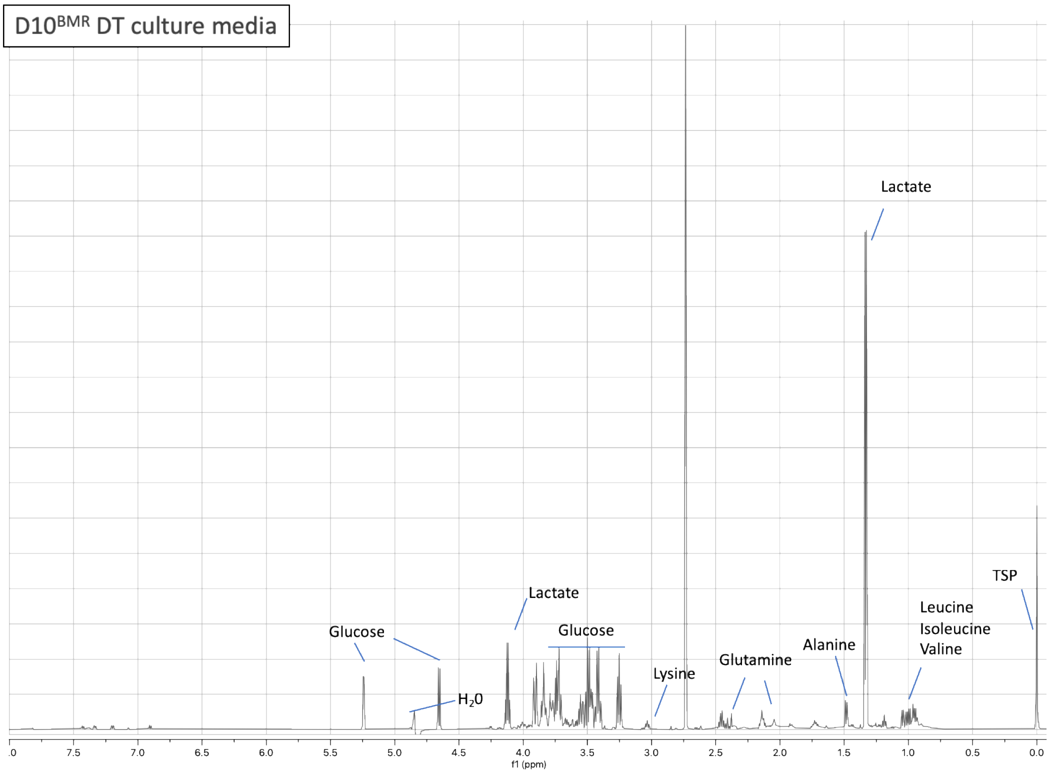

Figure S8. Intracellular (A) and extracellular (B) ^{1}H-NMR spectra of the MCF-7 cell line.

(A)

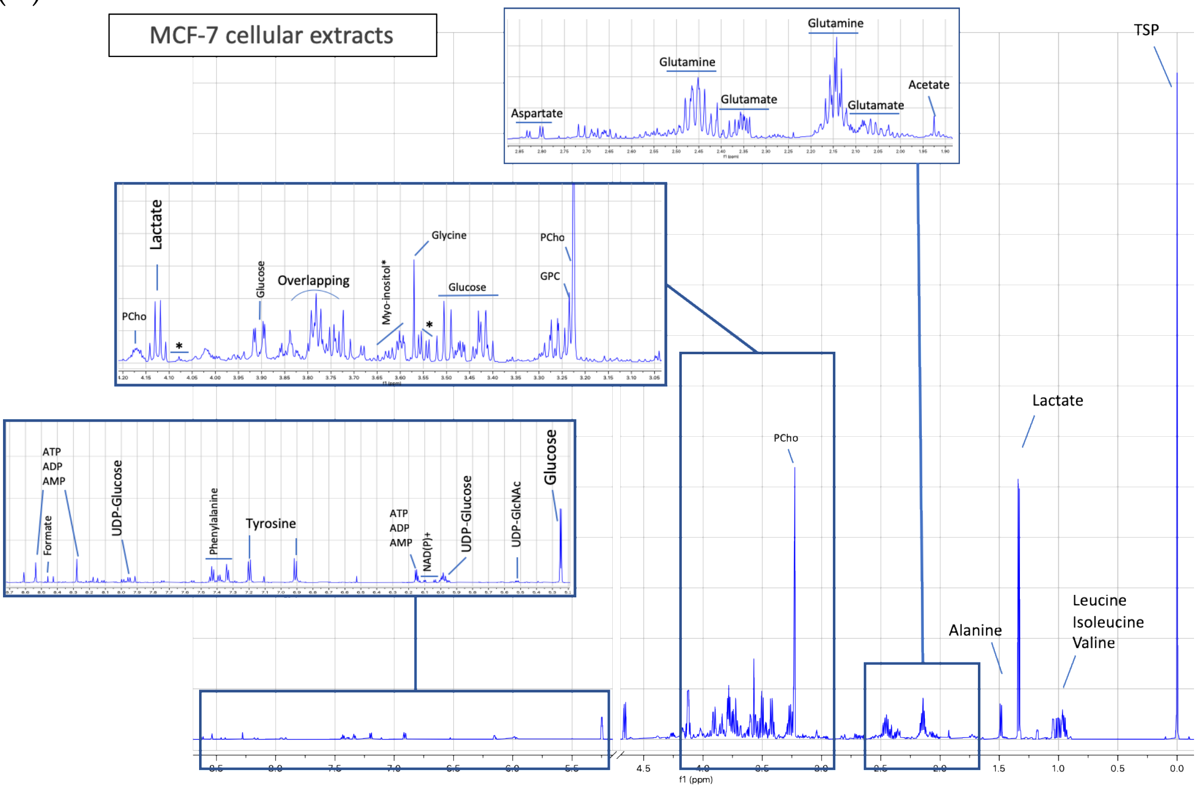

(B)

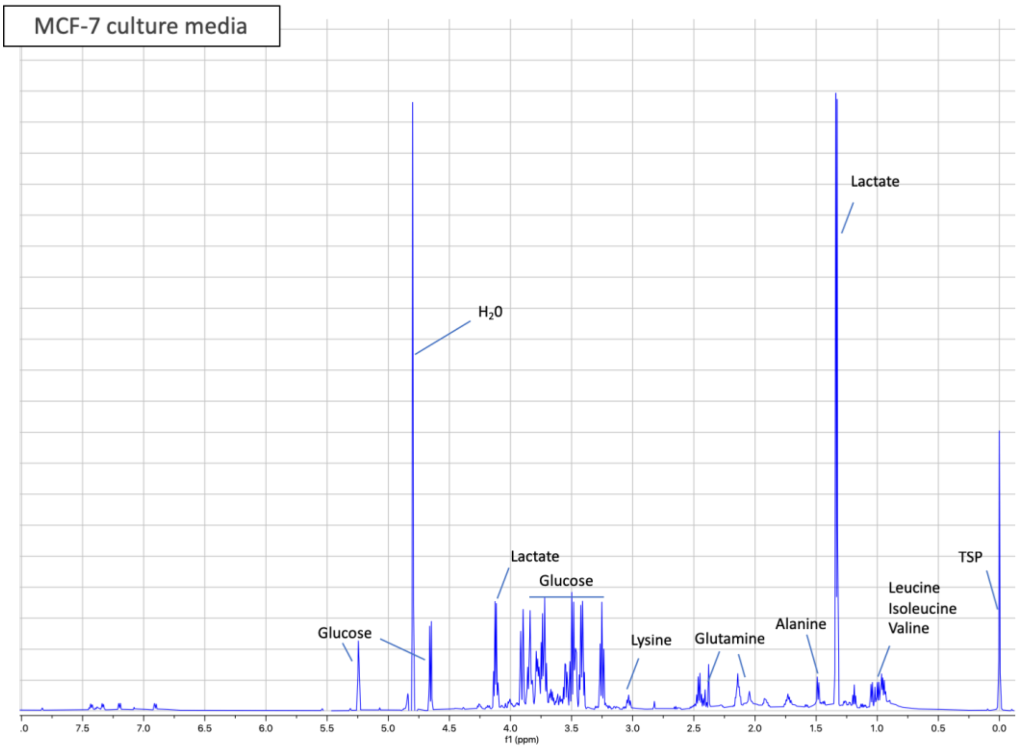

Figure S9. Intracellular (A) and extracellular (B) ^{1}H-NMR spectra of the MDA-MB-231 cell line.

(A)

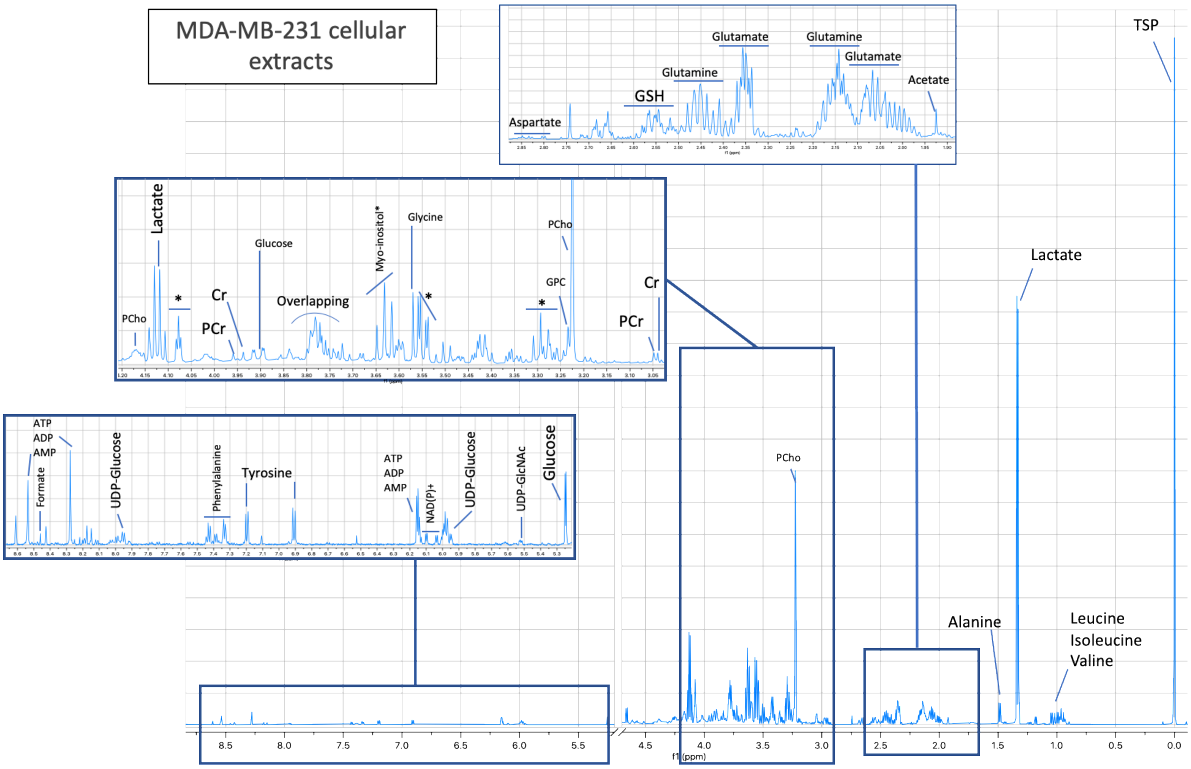

(B)

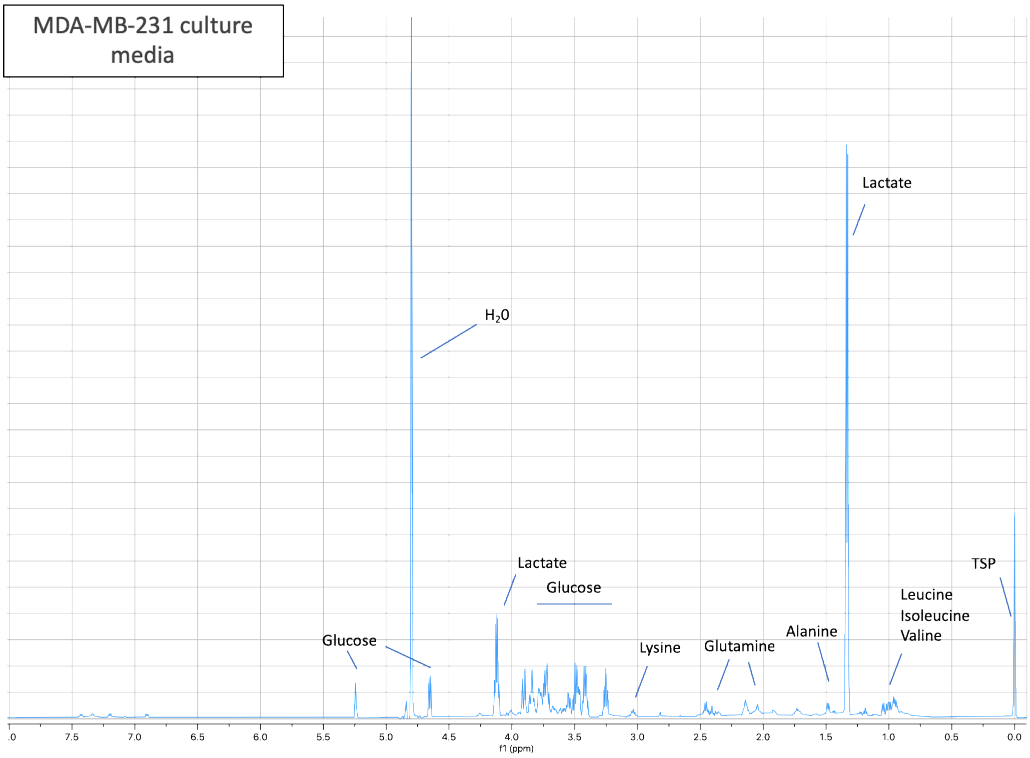

Figure S10. Intracellular (A) and extracellular (B) ^{1}H-NMR spectra of the PC-3 cell line.

(A)

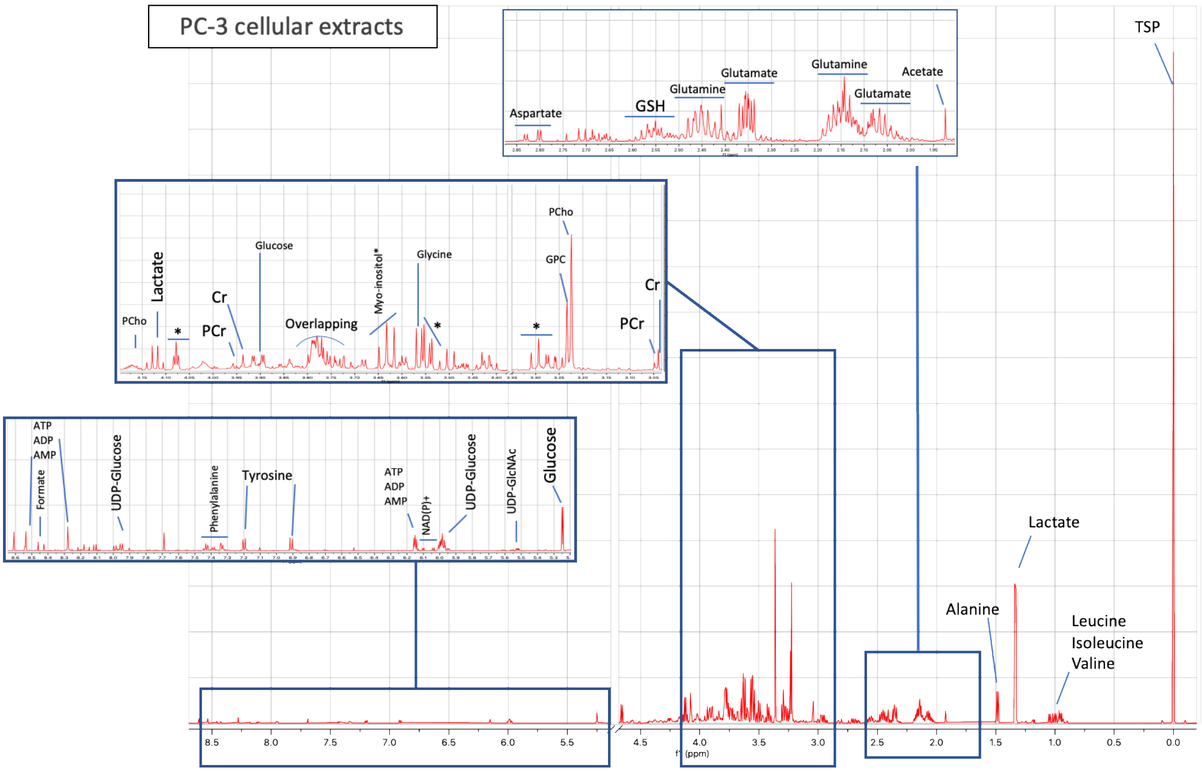

(B)

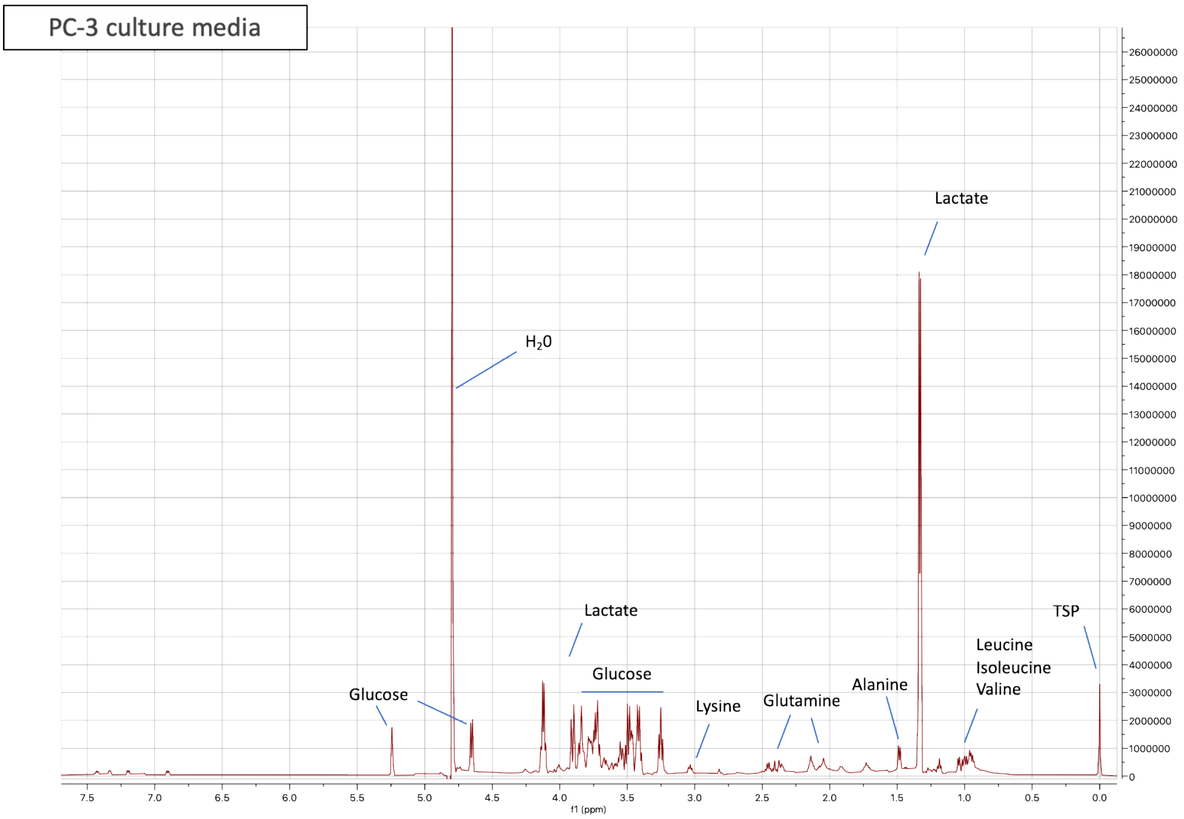

Figure S11. Intracellular (A) and extracellular (B) ¹H-NMR spectra of the LNCaP cell line.

(A)

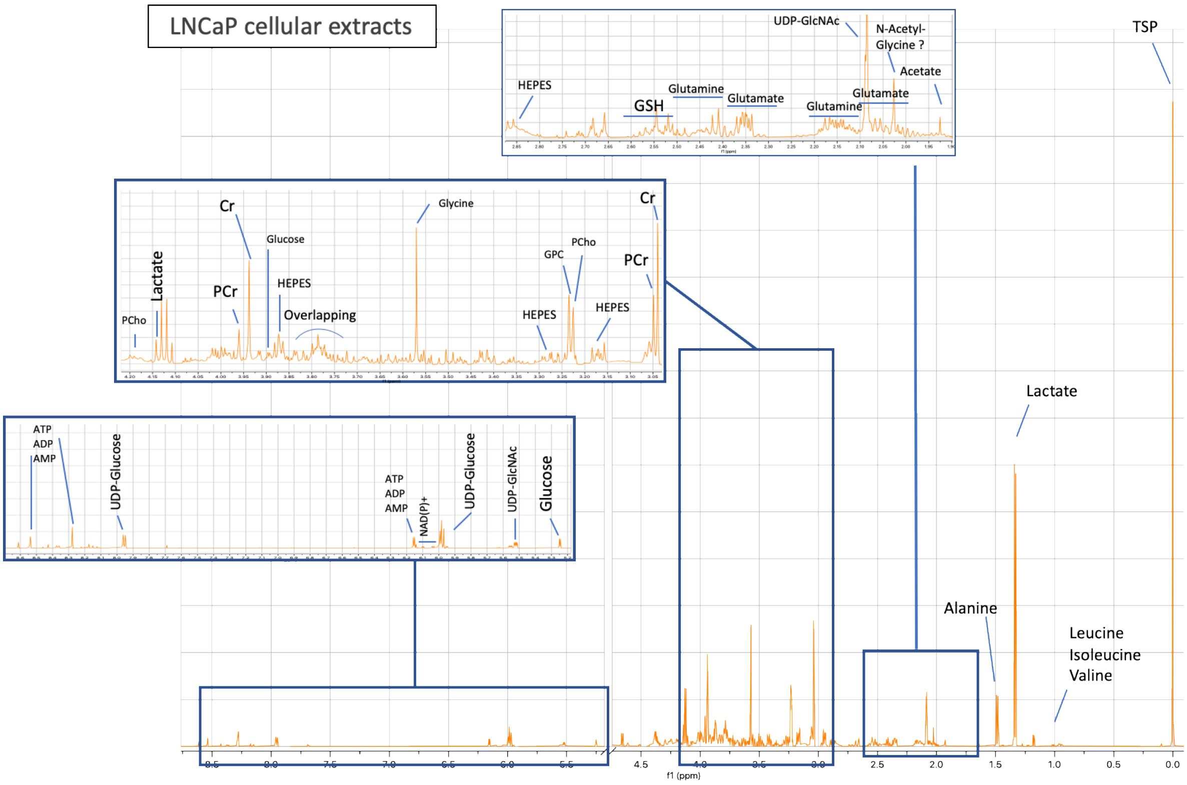

(B)

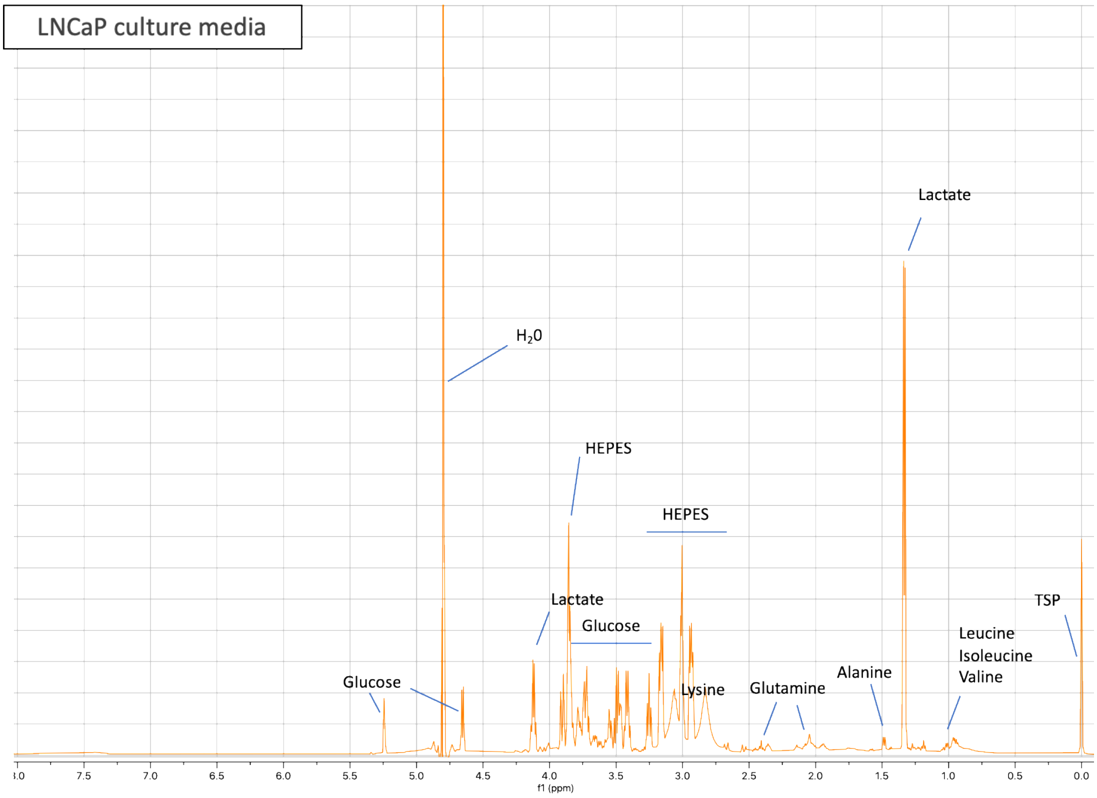

Supplement: Supplementary file 1 [file metabolites-09-00281-s001.pdf]
